# Deficient Beta-Mannosylation of *Candida albicans* Phospholipomannan Affects the Proinflammatory Response in Macrophages

**DOI:** 10.1371/journal.pone.0084771

**Published:** 2013-12-19

**Authors:** Audrey Devillers, Flavie Courjol, Chantal Fradin, Agnes Coste, Daniel Poulain, Bernard Pipy, Emerson Soares Bernardes, Thierry Jouault

**Affiliations:** 1 Inserm U995, Team 2, Lille, France; 2 Université Lille Nord de France, Lille, France; 3 Université Droit et Santé Lille2, Lille, France; 4 Unité Mixte de Recherche MD3, EA2405 Polarisation des macrophages et recepteurs nucleaires dans les pathologies inflammatoires et infectieuses, Université Paul Sabatier, Toulouse, France; 5 Unité mixte de recherche 152, Université Paul Sabatier, Toulouse, France; 6 Cancer Institute of the State of São Paulo, São Paulo, Brazil; Institut National de la Santé et de la Recherche Médicale U 872, France

## Abstract

*Candida albicans* produces a complex glycosphingolipid called phospholipomannan (PLM), which is present on the cell-wall surface of yeast and shed upon contact with host cells. The glycan moiety of PLM is composed of β-mannosides with degrees of polymerization up to 19 in *C. albicans* serotype A. PLM from serotype B strains displays a twofold decrease in the length of the glycan chains. In this study we compared the proinflammatory activities of PLMs purified from *C. albicans* serotype A and serotype B strains and from a *bmt6*Δ mutant of *C. albicans*, whose PLM is composed of short truncated oligomannosidic chain. We found that PLMs activate caspase-1 in murine macrophage cell line J774 independent of the glycan chain length although IL-1β secretion is more intense with long glycan chain. None of the tested PLMs stimulate ROS production, indicating that caspase-1 activation may occur through a ROS-independent pathway. On the other hand, only long-chain oligomannosides present on PLM from serotype A strain (PLM-A) are able to induce TNF-α production in macrophages, a property that is not affect by blocking endocytosis through latrunculin A treatment. Finally, we demonstrate that soluble and not cell surface-bound galectin-3, is able to potentiate PLM-A-induced TNF-α production in macrophages. PLMs from *C. albicans* serotype B and from *bmt6*∆ mutant are not able to induce TNF-α production and galectin-3 pretreatment does not interfere with this result. In conclusion, we show here that PLMs are able to evoke a proinflammatory state in macrophage, which is in part dependent on their glycosylation status. Long-glycan chains favor interaction with soluble galectin-3 and help amplify inflammatory response.

## Introduction

*Candida albicans* produces a complex glycosphingolipid (GSL) called phospholipomannan (PLM) [1], which is present at the surface of the cell wall and is shed in contact with host cells [2-5]. Although the impact of PLM on *C. albicans* biology is unknown, it is considered to be a virulence factor of *C. albicans*. PLM induces tumor necrosis factor alpha (TNF-α) secretion from cells of macrophage lineage via a TLR-2-dependent pathway [6,7] and macrophage apoptosis via modulation of the ERK pathway [8,9] induced at the time of phagocytosis. The glycan moiety of PLM is unique. It is mainly composed of β-1,2 mannosides with degrees of polymerization up to 19 in *C. albicans* serotype A strains. PLM from serotype B strains displays shorter β-1,2 mannosides chains with a predominant mannotriose [10]. β-1,2 mannosides by themselves act as stimuli for macrophage activity and are recognized by macrophages through an interaction involving galectin-3, a glycan-binding protein highly produced and secreted by macrophages [11]. Therefore, due to the prominent cell surface expression of β-1,2 mannosides in *C. albicans*, galectin-3 is able to discriminate between this pathogen [12] and the non-pathogenic yeast *Saccharomyces cerevisiae* [13,14]. 

It has been shown that the complete absence of PLM affects the virulence of *C. albicans* [15]. Alteration of the *MIT1* gene in *C. albicans* resulted in complete absence of glycosylated PLM. Absence of *MIT1* leads to no biosynthesis, then no secretion of PLM, and abolishes the immune activities of yeasts both *in vitro*, toward macrophages, and in animal models [15]. The absence of PLM caused by deletion of *MIT1* led to a decrease in virulence of yeasts during both the acute and chronic phases of systemic infection in mice (75 and 50% reduction in mortality, respectively). *In vitro*, *mit1*Δ mutant was not able to escape macrophage lysis through downregulation of the ERK1/2 phosphorylation, a previously shown PLM-induced escape mechanism [15].

 Recently, a new family of genes encoding β-mannosyltransferases (Bmt1–9) has been identified as responsible for adding β-1,2 mannosides to different carriers present in the *C. albicans* cell wall [16]. Among these nine β-mannosyltransferases, Bmt6 was shown to be one of the enzymes specifically involved in β-1,2 mannosylation of PLM. Deletion of *BMT6* results in an impairment in the addition of the third β-mannose to PLM backbone [10,17] but has no impact on upstream sphingolipid (IPCs, MIPCs and M (IP)_2_Cs) biosynthesis, meaning that both wild-type (WT) and *bmt6*∆ mutant yeasts share a common lipid backbone. In addition, it does not affect the addition of β-mannose to other carriers in *C. albicans* such as phosphopeptidomannan and mannoproteins.

 The aim of this study was to investigate the effects of differential β-1,2 mannosylation of PLM on its ability to induce proinflammatory response in macrophages. Therefore, we compared the proinflammatory properties of three different types of PLMs: PLM-A, isolated from SC5314, a WT *C. albicans* serotype A strain; PLM-B, isolated from the NIHB strain, a serotype B strain and PLM-BMT6∆, isolated from the *bmt6*∆ mutant. We conclude that PLMs displaying short and truncated glycan chains are still able to lead to inflammasome activation in macrophage, indicating that PLM lipid backbone plays a role in this process. However, the presence of long chains of β-1,2-linked mannosides is essential for the induction of TNF-α production in macrophages and the binding of PLM-A to soluble galectin-3, which may amplify PLM-A-induced inflammatory response.

## Materials and Methods

### Reagents and antibodies

 All reagents were obtained from Sigma Aldrich (Saint-Quentin Fallavier, France). Fluorochrome inhibitor of caspase 1 (FLICA) was purchased from Immunochemistry Technologies (Bloomington, MI). Anti-caspase-1 p10 antibody (M-20) was obtained from Santa Cruz Biotechnology (Santa Cruz, CA). The anti-galectin-3 antibody (clone M3/38) was purchased from Millipore (Temecula, CA). FITC-conjugated anti-mouse IgG antibody and horseradish peroxidase (HRP)-conjugated anti-rabbit IgG were obtained from Southern Biotechnology Laboratories (Birmingham, AL). 

 To avoid possible LPS contamination, which could interfere with the observations, purified PLMs and r-Gal3 were tested for endotoxin contamination (Limulus Amebocyte Lysate - Pyrogent Plus, Lonza - Walkersville, MA). Endotoxins were below the detection limit of the assay (<0.03 EU/ml). 

### Strains and Growth Conditions

*C. albicans* wild type strains SC5314 (serotype A, [18]) and NIH B 792 (serotype B, [19]) were used in this study. The mutant strain, *bmt6*Δ, expressing a truncated PLM was obtained after deletion of *BMT6* from strain BWP17 by PCR based gene targeting [20].

 Yeast cells were maintained at 4°C on YPD (1% yeast extract, 2% peptone, 2% dextrose)-agar medium. Before each experiment, yeast cells were transferred onto fresh YPD and incubated overnight at 37°C. Yeasts were recovered by centrifugation, resuspended in phosphate buffered saline (PBS) and used at different concentrations.

### PLMs purification

 PLM from the different strains was purified as described previously [1]. Sphingolipids were first extracted from dried yeasts with chloroform/methanol/water (10/10/3) solution. After extensive butanol/water partitions, aqueous components, among which PLM, were purified on phenyl-sepharose using increasing concentrations of ethanol (1–40%) for elution. Purified PLM fractions were checked and analyzed by thin-layer chromatography using a butanol/acetic acid/water (20/8/17) solvent system and visualized with orcinol reagent. β-1,2 mannosides released from the different PLMs were analyzed by Fluorescent Assisted Carbohydrate Electrophoresis (FACE). 

### Fluorescent Assisted Carbohydrate Electrophoresis

 Purified PLMs were hydrolyzed in 20 mM HCl for 1 h at 100°C to release β-1,2 oligomannosides. After neutralization, hydrolysates were dried before to be tagged with 0.15 M 8-amino-naphthalene-1,3,6-trisulfonate (ANTS) and 1 M sodium cyanoborohydride for 16 h at 37°C [21]. The dried samples were resuspended in glycerol/water (1/4). Electrophoresis of ANTS-labeled oligomannosides was performed on 35% (w/v) acrylamide separating gels. Acid-hydrolyzed dextran and purified oligosaccharides were used as carbohydrate standards. Gels were visualized with a 365 nm UV-transilluminator.

### Cell lines

 The mouse macrophage-like J774 cell line (ECAC 85011428), derived from a tumor of a female BALB/c mouse, was from the European Collection of Cell Cultures (ECAC) and is provided by Sigma Aldrich. Adherent cells were cultured at 37°C in an atmosphere containing 5% CO2 in DMEM supplemented with 10% heat-inactivated fetal calf serum (FCS) (Valbiotech, Paris, France), 5 mM L-glutamine, 100 µg/ml streptomycin, and 50 µg/ml penicillin (culture medium). Before use, the cells were gently scraped off with a rubber policeman and, depending on the experiment, either plated onto 8-well Labtek tissue culture chambers (Nunc, Naperville, IL) or onto 24- or 48-well tissue culture dishes.

### Stimulation of cells with PLMs

 Macrophages (5x10^5^cells/well) were incubated with different concentrations of PLMs for 3 or 5h at 37°C for TNF-α production. For IL-1β production, platted cells were primed for 5h with 500 ng/ml of LPS and then incubated with PLMs for 12 or 24h. After incubation, the supernatants were collected and stored at -80°C until cytokine assays were performed. Cytokine concentrations in cell-free supernatants were measured using a commercial ELISA kit according to the manufacturer’s instructions (eBioscience, San Diego, CA). In some experiments, plated cells were incubated for 30 min with 2 ng/ml Latrunculin A (Cayman Chemical, Ann Arbor, MI) prior addition of PLMs in order to inhibit activation depending on internalization.

### Galectin-3 preparation

 Recombinant human Galectin-3 (rGal-3) was produced in *Escherichia coli* and prepared as previously described [22]. rGal-3 was purified by affinity chromatography and detoxified on detoxi-gel beads (Detoxi-Gel Endotoxin Removing Columns, Thermo-Fisher Scientific - Rockford, IL) to eliminate any contaminating LPS. Endotoxin contamination tested with the Limulus Amebocyte Lysate, Pyrogent Plus (Lonza) was below the detection limit of the assay. 

### Binding of rGal-3 to cells

 Macrophages (5x10^5^cells/well) platted onto 8-well Labtek tissue culture chambers were incubated with rGal-3 at 10 µg/ml for 1 h. Binding of rGal-3 was stained with the anti-Gal3 antibody (Clone M3/38) and FITC anti-mouse IgG (Southern Biotech). After fixation with 4% paraformaldehyde in PBS, the slides were mounted and the cells were examined by fluorescent microscopy.

### Effect of rGal-3 on stimulation

 Macrophages (5x10^5^cells/well) plated onto 48-well tissue culture dishes were incubated with rGal-3 at a concentration of 10 µg/ml for 1 h. Cells were then stimulated for 3 to 5 h with or without different concentrations of PLM or with Pam3CSK4 (500 ng/ml) or curdlan (100 and 10 µg/ml) used as control. In parallel experiments, PLM or control stimuli were incubated for 1 h at 37°C with rGal-3 (10 µg/ml) before addition to the cells. 

PLM and control stimuli were incubated with or without 10 µg/ml of rGal-3 for 1 h at 37°C. The mixture was then added to plated cells as described above for stimulation. In parallel, PLM and control stimuli were incubated for 1 h with rGal-3 (10 µg/ml) before addition to the cells. rGal-3 activity was inhibited by incubation with 50 mM lactose, a specific inhibitor of Galectin-3, for 15 min before the experiments.

### Reactive oxygen species (ROS) production by cells

 The oxygen-dependent respiratory burst of cells was measured by chemiluminescence in the presence of 5-amino-2,3-dihydro-1,4-phthalazinedione (66 µM, Luminol; Sigma-Aldrich) using a thermostatically (37°C) controlled luminometer (Wallac 1420 Victor2). The production of chemiluminescence was monitored continuously for 2 h after cell stimulation with yeast cells (5:1, yeast:cell ratio) or PLMs. One hundred µM of 12-O-tetradecanoyl-phorbol-13-acetate (TPA) was used as a positive control for stimulation. Inhibition of ROS production was performed 30 min before stimulation using Latrunculin A (500 ng/ml).

### Caspase 1 activation

 After plating in Labtek tissue culture chambers, macrophages (3x10^5^ cells/well) were incubated with lipopolysaccharide (LPS) (100 ng/ml) for 2 h before addition of PLMs (50 µg/ml), aluminum hydroxide (Alum, 0.1 µg/ml) or ATP (25 µg/ml), for 4 hours. Cells were then treated either for proteins extraction for Western blot analyses or for detection of caspase-1 activation. 

### Western blot analysis of activated caspase-1

 Total cell proteins were extracted with RIPA buffer (1% NP40, 0.5% Sodium Deoxycholate, 0.1% SDS) for 30 min on ice with occasional checking. Lysates were clarified by centrifugation for 10 min at 12 000 x *g* at 4°C and then conserved at -20°C. A fixed amount of lysate, determined by the protein concentration, was mixed with 4 x concentrated electrophoresis sample buffer (1 x : 125 mM Tris-HCl, pH 6.8, 2% SDS, 5% glycerol and bromophenol blue) and boiled for 5 min at 100°C. The extracted proteins were then separated by 15 % SDS polyacrylamide gel electrophoresis and transferred to a nitrocellulose membrane (Schleicher and Schuell, Dassel, Germany) for 1 h at 200 mA in a semi-dry transfer system. After staining with 0.1% Ponceau S in 5% acetic acid to confirm equivalence of loading and transfer, membrane were saturated with TNT (Tris 10 mM, NaCl 100 mM, Tween 0.1%) containing 5% non-fat dry milk for 1 h at 20°C. The membrane was then probed with anti-caspase-1 p10 (M-20) (diluted 1:1000) in TNT-0.5% non-fat dry milk overnight at 4°C. After several washes, the membrane was incubated for 2 h at 20°C with a 1:1000 dilution of HRPO-conjugated anti-rabbit IgG in TNT-0.5% non-fat dry milk. Finally, proteins were visualized with ECL detection reagents (ThermoFisher, Whaltman, MA) and exposed to hyperfilm ECL.

### Fluorescent examination of activated caspase-1

 Macrophages (3x10^5^ cells/well) were platted overnight onto 8-wells Labtech chambers (Nunc). Cells were then stimulated with LPS for 2h. Stimuli were then added and incubation continued for 4 h. After washing, the cells were stained with the fluorescent specific inhibitor of caspase-1, FAM-YVAD-FMK (5-carboxyfluorescein–Tyr-Val-Ala-Asp–fluoromethylketone, FLICA) according to the manufacturer’s instructions (Immunochemistry Technologies, Bloomington, MN). The slides were then washed and mounted for microscopic examination. 

### Statistical analysis

 All experiments were repeated at least three times. For cytokine production, the values reported are the means ± SD of the results obtained from three independent experiments. Statistical significance was accepted at *P*<0.05, determined by ANOVA or student's t test depending on the experiments.

## Results

### PLMs activate inflammasome in a glycan-independent manner

**Figure 1:**
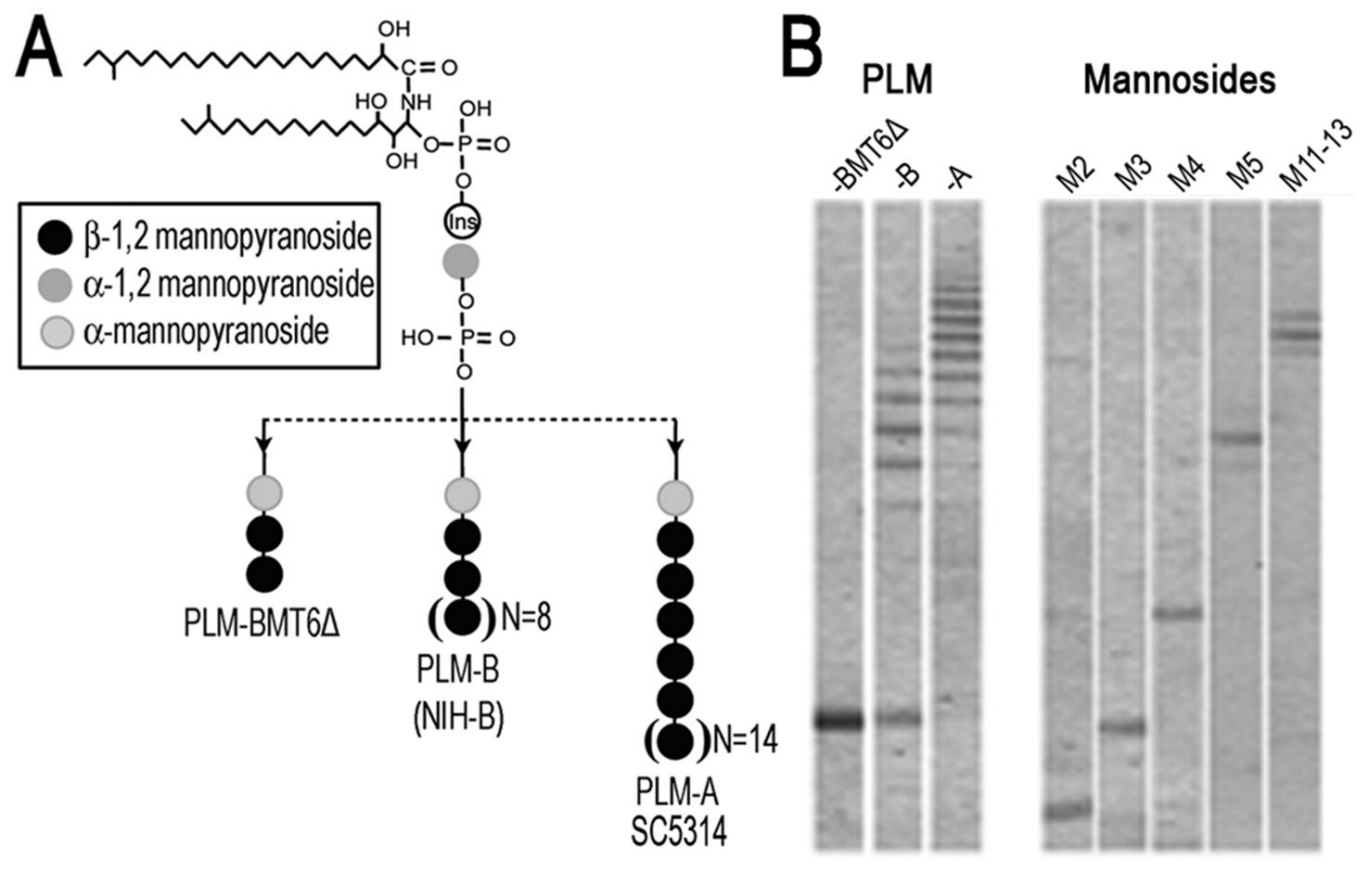
Structural comparison between glycan chains of PLM-A, PLM-B and PLM-BMT6Δ. (**A**). General structure of PLMs used in the present study, showing the differences in glycan chain length linked to the lipid backbone. (**B**). PLM-A was isolated from SC5314, a WT *C. albicans* serotype A strain; PLM-B, isolated from the NIHB strain, a serotype B strain and; PLM-BMT6∆, isolated from the *bmt6*∆ mutant. Following, PLMs were submitted to hydrolysis and analyzed by Fluorophore-assisted carbohydrate electrophoresis (FACE) method. The analysis shows the different mannosidic chains released after the hydrolysis protocol. Isolated mannosides (M2, M3, M4, M8, M11-13) were used as control.

 In order to investigate the effects of PLM glycosylation on macrophage activation, we first purified PLMs from *C. albicans* serotype A (SC5314), *C. albicans* serotype B (NIHB) and from the *bmt6*∆ mutant of *C. albicans*. Molecular structural differences between the three different types of PLMs are summarized in the Figure 1A. Fluorophore-assisted carbohydrate electrophoresis (FACE) analyses revealed the major oligomannosides released by acid hydrolysis of PLMs (Figure 1B). As can be seen in the Figure 1 (A and B), PLM from serotype A strains (PLM-A) display a twofold increase in glycan chain length in comparison with PLM from serotype B (PLM-B), and PLM from *bmt6*∆ mutant (PLM-BMT6∆) which displays a short, truncated glycan chain.

**Figure 2:**
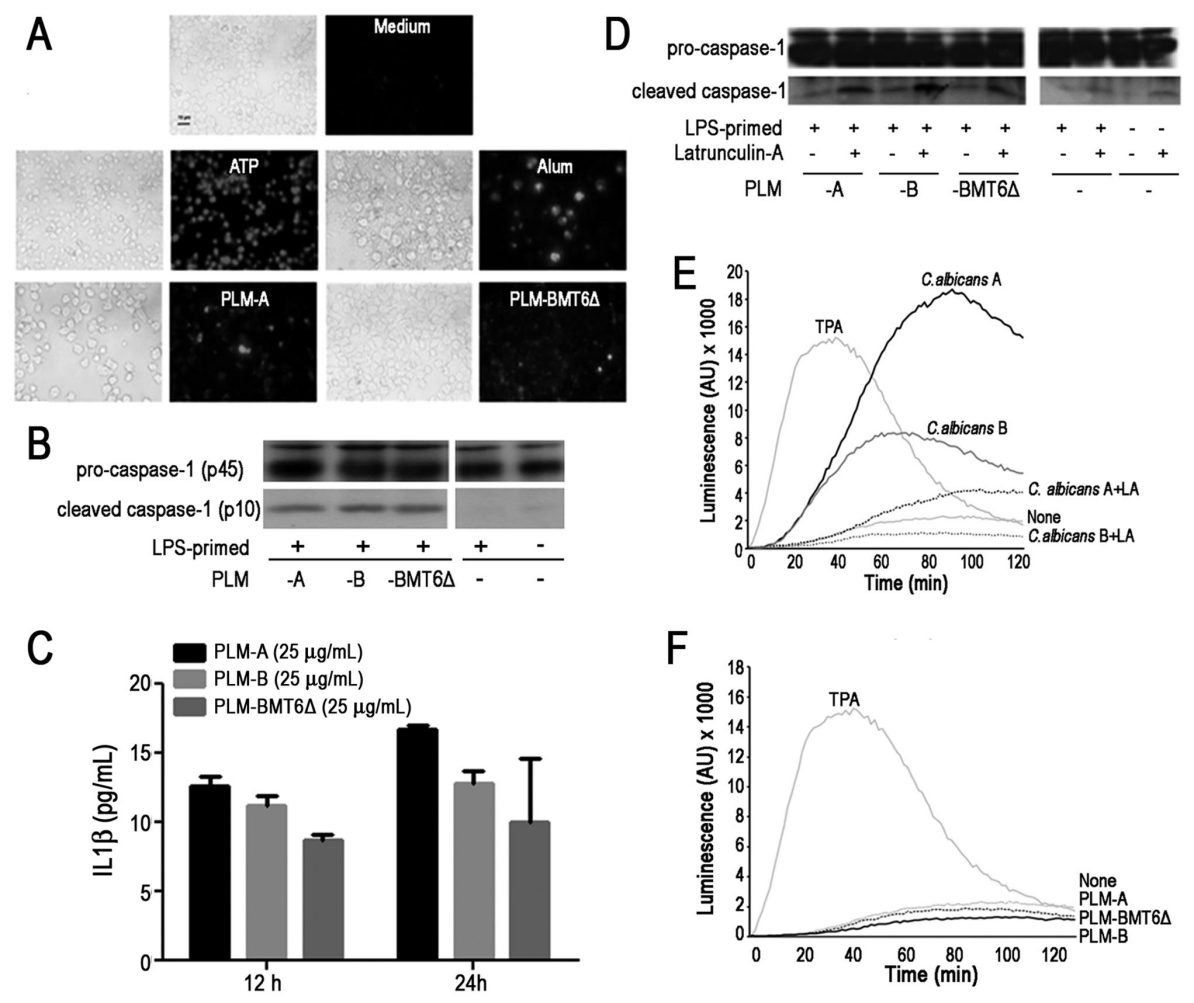
PLMs induce caspase-1 activation independent on ROS production. (**A**-**C**). J774 macrophages were primed with LPS (100 ng/ml) for 2h. Then, indicated stimuli were added and cells were cultivated for additional 4h. (**A**). Caspase-1 activation was revealed by addition of fluorescent FAM-YVAD-FMK and examined by fluorescent microscopy. Adenosine triphosphate (ATP) and Aluminum hydroxide (Alum) were used as positive control for caspase-1 activation. (**B**). Total pro-caspase-1 and cleaved caspase-1 levels were revealed by western blot analysis. (**C**). Production of IL-1β after stimulation with PLMs of LPS-primed cells was measured after 12- and 24h-incubation by ELISA. (**D**). Macrophages were pretreated or not with Latrunculin-A and then stimulated with indicated PLMs. Total pro-caspase-1 and cleaved caspase-1 levels were revealed by western blot analysis. Data are representative of 4 independent experiments. (**E** and **F**). Macrophages were pretreated or not with Latrunculin A for 30min. Live yeast cells at 5 yeast: 1 cell ratio (**E**) or PLMs (50µg/ml) (**F**) were then added. Chemiluminescence was monitored for 2h in the presence of 66 µM of 5-amino-2,3-dihydro-1,4-phthalazinedione (luminol). 12-O-tetradecanoylphorbol-13-acetate (TPA, 100 µM) was used as a positive control. Results are representative of 2 independent experiments.

 Because inflammasome plays an essential role in antifungal immunity, we tested whether PLMs with different levels of glycosylation were able to induce caspase-1 activation in macrophage. Then, cells were primed with LPS and stimulated with PLMs, ATP or Alum (used as a positive controls for caspase-1 activation) and the cleavage of pro-caspase-1 into active caspase was examined by FLICA (Fluorescent Labeled Inhibitor of Caspases) assay. As shown in the Figure 2A, PLMs (either PLM-A or PLM-BMT6∆) were able to activate caspase-1. To further confirm this result, LPS-primed macrophages were stimulated with PLMs and the activation of caspase-1 was monitored by Western blot. The active caspase-1 (p10) was detected in cell lysates of PLMs-stimulated macrophage, irrespective of their glycosylation status (Figure 2B). As expected, LPS-primed murine macrophages did not exhibit significant caspase-1 activation without secondary stimulation (Figure 2B).

 To further examine the effect of PLMs on inflammasome activation, cells were primed with LPS and IL-1β production was evaluated in cell-free supernatants. As shown in the Figure 2C, PLM-A was able to induce the secretion of IL-1β by primed cells. No cytokine was produce by unprimed cells (data not shown). PLM-B and PLM-BMT6∆ also induced the secretion of IL-1β by primed cells, although the amount of cytokine was lower compared to that obtained with PLM-A.

 In order to determine whether PLMs must be internalized to induce the activation of caspase-1, LPS-primed macrophages were treated with a known inhibitor of endocytic activity, Latrunculin A. Interestingly, Latrunculin A was not able to inhibit caspase-1 activation induced by any of the tested PLMs (Figure 2D). Altogether, these results demonstrate that PLMs induce inflammasome activation and IL-1β production in LPS-primed macrophages.

### PLMs Are Not Able to Induce ROS Production in Macrophage

 Since reactive oxygen species (ROS) have been shown to be one of the main mechanisms involved in caspase-1 activation [23], the effect of purified PLMs on ROS stimulation was also investigated. First, we checked whether *C. albicans* yeast cells of serotype A and B display any difference in their capacity to induce ROS generation in macrophage. As shown in the Figure 2E, although both *C. albicans* serotype A and B were able to stimulate the production of ROS by macrophages, *C. albicans* serotype A induced higher levels of ROS even in comparison with TPA, used as a positive control. In both cases, ROS generation was inhibited by pretreatment with Latrunculin A, indicating that phagocytosis of the yeasts was required for the induction of ROS production by macrophages. In contrast to *C. albicans* yeast cells, all tested PLMs did not induce any ROS stimulation (Figure 2F). This result suggests that PLMs-induced caspase-1 activation is not linked to ROS generation.

### Long-chain oligomannosides of PLM are required for induction of TNF-α in macrophage

**Figure 3:**
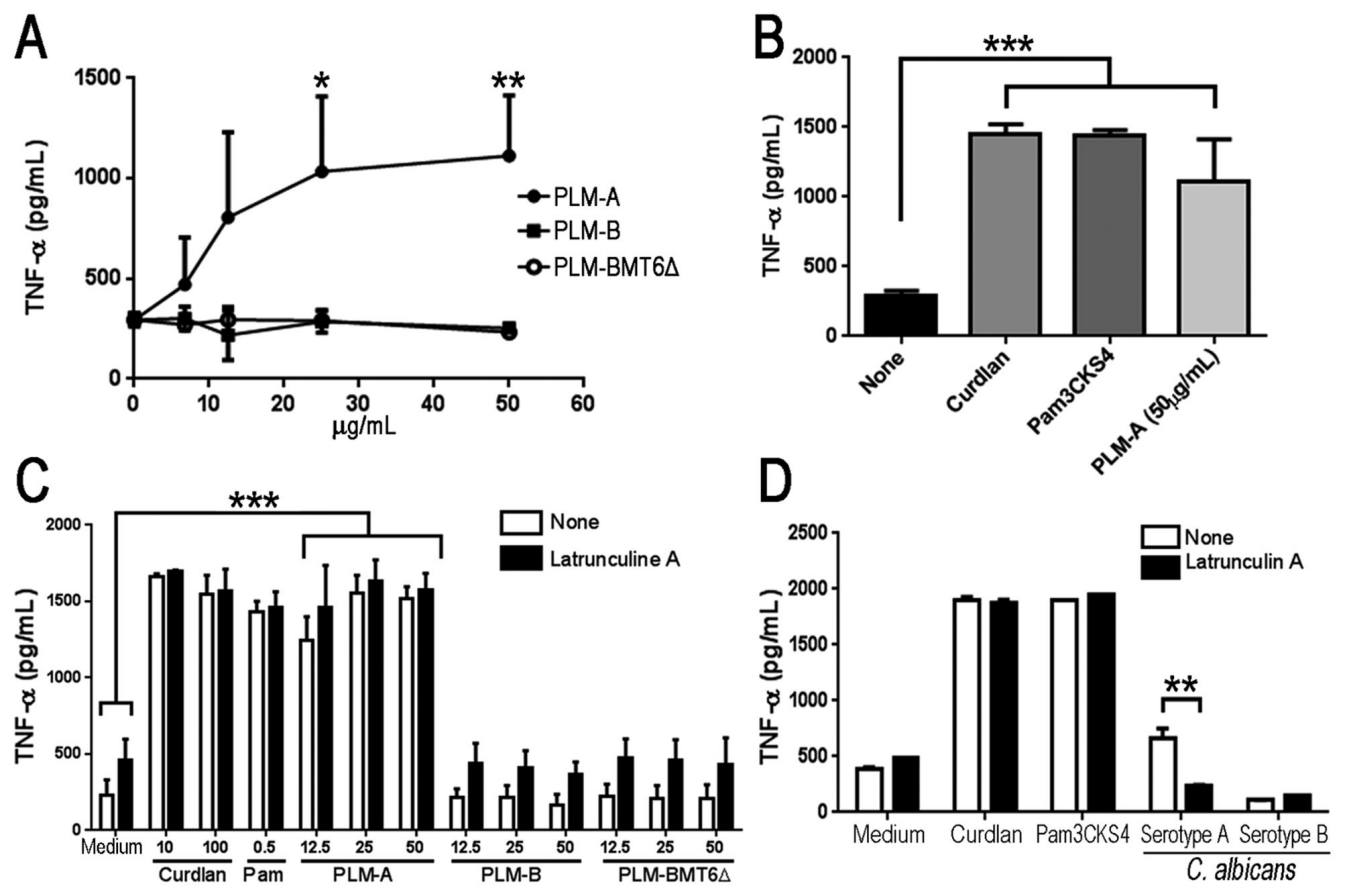
Long chain oligomannosides are required for PLM-induced TNF-α production. (**A**). J774 macrophages were incubated with increasing concentration of PLM-A (●), PLM-B (■) or PLM-BMT6∆ (○) and cultivated for 5h at 37°C. Cell culture supernatants were harvested and TNF-α production was evaluated by ELISA. (**B**). Alternatively, cells were stimulated with PLM-A (50 µg/ml) or with curdlan (100 µg/ml) and Pam3CSK4 (500 ng/ml) as positive controls. Data represents the mean ± standard deviation from 3 independent experiments, each one performed in triplicates. (**C** and **D**). J774 macrophages were pretreated (■) or not (□) with Latrunculin A for 30 min prior to PLMs addition at the indicated concentrations (**C**) or live *C. albicans* serotype A and B yeast cells at a 5 yeast : 1 cell ratio (**D**). Curdlan and Pam3CSK4 were used as positive controls. TNF-α concentration in cell-free supernatants was determined by ELISA after 4h incubation. Data are representative of two independent experiments performed in triplicates. *p<0.05; **p<0.01; ***p<0.0005.

 We have previously shown that PLM is able to stimulate TNF-α production by macrophage in a Toll-like receptor-dependent manner [13]. Because TNF-α production occurs via a distinct pathway independent of inflammasome and caspase-1 activation, we also checked whether or not glycosylation changes in PLMs would affect their ability to induce TNF-α production. The PLMs isolated from the different sources were first examined for their ability to stimulate TNF-α production by macrophages. As expected [6,13], PLM-A isolated from strain SC5314 (which displays long-chain oligomannosides) was able to induce TNF-α production in a dose-dependent manner and reached a plateau at a PLM-A concentration of 25µg/ml (Figure 3A). In contrast, macrophages incubated with PLM-B purified from *C. albicans* serotype B (which displays oligomannoside-type chains of intermediate size) or with PLM-BMT6∆ from yeast mutant lacking *BMT6* gene (which displays a short truncated glycan chain) did not stimulate significant TNF-α production even at the highest dose tested (Figure 3A). In addition, PLM-A induced levels of TNF-α similar to those observed with 100µg/ml of curdlan or 500 ng/ml of Pam3CSK4, used as a positive control for dectin-1 and TLR2 activation, respectively (Figure 3B). These results suggest that the glycan chain in PLMs plays an essential role in inducing TNF-α production in macrophages.

### PLM-induced secretion of TNF-α is independent of internalization

 Taking into consideration that the molecular differences between the three different types of PLMs (Figure 1A) may affect their intermolecular interactions and lead to a different pattern of internalization and/or interaction with cell surface receptors, we next tested whether Latrunculin A would affect PLM-induced TNF-α. As shown in the Figure 3C, PLM-A was the only molecule able to induce TNF-α production and the pretreatment of macrophages with Latrunculin A had no effect on its capacity to induce TNF-α. As expected, PLM-B and PLM-BMT6∆ did not stimulate TNF-α production and this result was not changed by pretreatment of the macrophages with Latrunculin A (Figure 3C). Similar to PLM-A, neither curdlan nor Pam3CSK4-stimulated macrophages were affected by pretreatment with Latrunculin A, demonstrating that internalization had no effect on the response to these soluble stimuli (Figure 3D). On the other hand, macrophage pretreatment with Latrunculin A led to a significant decrease in TNF-α production induced by *C. albicans* yeast cells (serotype A), showing that phagocytosis is an important step for host defense against *C. albicans* (Figure 3D). *C. albicans* yeast cells (serotype B), which are similarly phagocytized by macrophages (data not shown), were unable to induce significant production of TNF-α, regardless of whether the macrophages were pretreated with Latrunculin A (Figure 3D). Taking together, these results demonstrate that PLM-A-induced TNF-α production is not dependent on internalization and may involve direct interaction of PLM-A with cell surface receptors.

### Galectin-3 interacts with long-chain oligomannosides of PLM and potentiates its activities on macrophage

**Figure 4:**
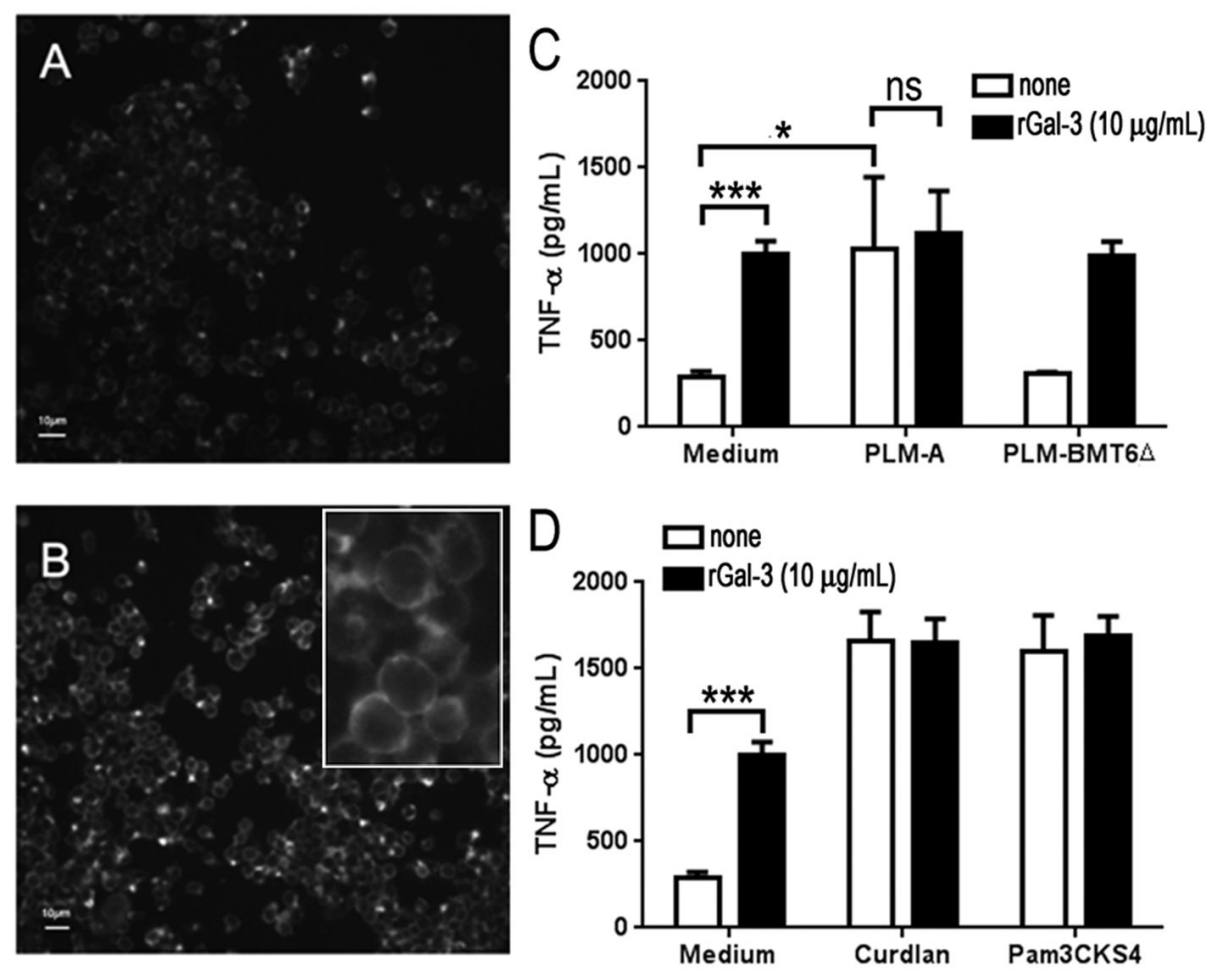
Cell-bound galectin-3 does not potentiate PLM-A-induced TNF-α production. (**A**). The levels of endogenous galectin-3 bound to the J774 cell surface were determined by immunofluorescent staining using anti-galectin-3 antibody and FITC-conjugated secondary antibody. Cells were then fixed and analyzed by fluorescence microscopy. (**B**). Alternatively, cells were incubated with 10 µg/ml of exogenous rGal-3 for 1h at 20°C before staining and analysis performed as described in (A). Insert shows high magnification of labeled cells. (**C** and **D**) Cells were preincubated for 1h with (■) or not (□) 10 µg/ml of exogenous rGal-3 prior to the addition of 10 µg/ml of PLM-A or PLM-BMT6∆ (**C**) 10 µg/ml of curdlan or 500 ng/ml of Pam3CSK4 (**D**). Cells were cultivated for additional 4h. Then, supernatants were collected and TNF-α concentration was determined by ELISA. Results are presented as the mean ± standard deviation from 3 independent experiments. *p<0.05; **p<0.01; ***p<0.0005.

 We have previously shown that galectin-3 acts as a receptor for β-1,2 linked oligomannosides [11], which in association with TLR2 allows macrophages to discriminate between pathogenic and non-pathogenic yeasts based on their cell surface glycans [13]. Therefore, we investigated whether changes in PLM glycosylation would affect its interaction with galectin-3 and interfere with the PLMs capability to stimulate macrophages. First we verified the galectin-3 expression and galectin-3-binding sites levels on the macrophage surface. As expected, J774 macrophages expressed low levels of endogenous galectin-3 (Figure 4A) and the addition of exogenous rGal-3 led to an enhanced level of galectin-3 at the cell surface (Figure 4B). Then, we checked whether the enhanced levels of galectin-3 at the cell surface would interfere with PLM-A-induced TNF-α production in macrophages. As shown in the Figure 4C, there was no significant difference in TNF-α production when rGal-3-pretreated macrophages were stimulated with PLM-A in comparison with PLM-A stimulation alone. PLM-BMT6∆ was not able to induce TNF-α even in rGal-3-pretreated macrophages, which displayed increased levels of TNF-α (Figure 4C). Thus, PLM-A had no additive or enhanced effect on rGal-3-pretreated macrophages. Similar results were obtained with curdlan or Pam3CSK4, both of which induced similar levels of TNF-α in rGal-3-pretreated or not macrophages (Figure 4D).

**Figure 5:**
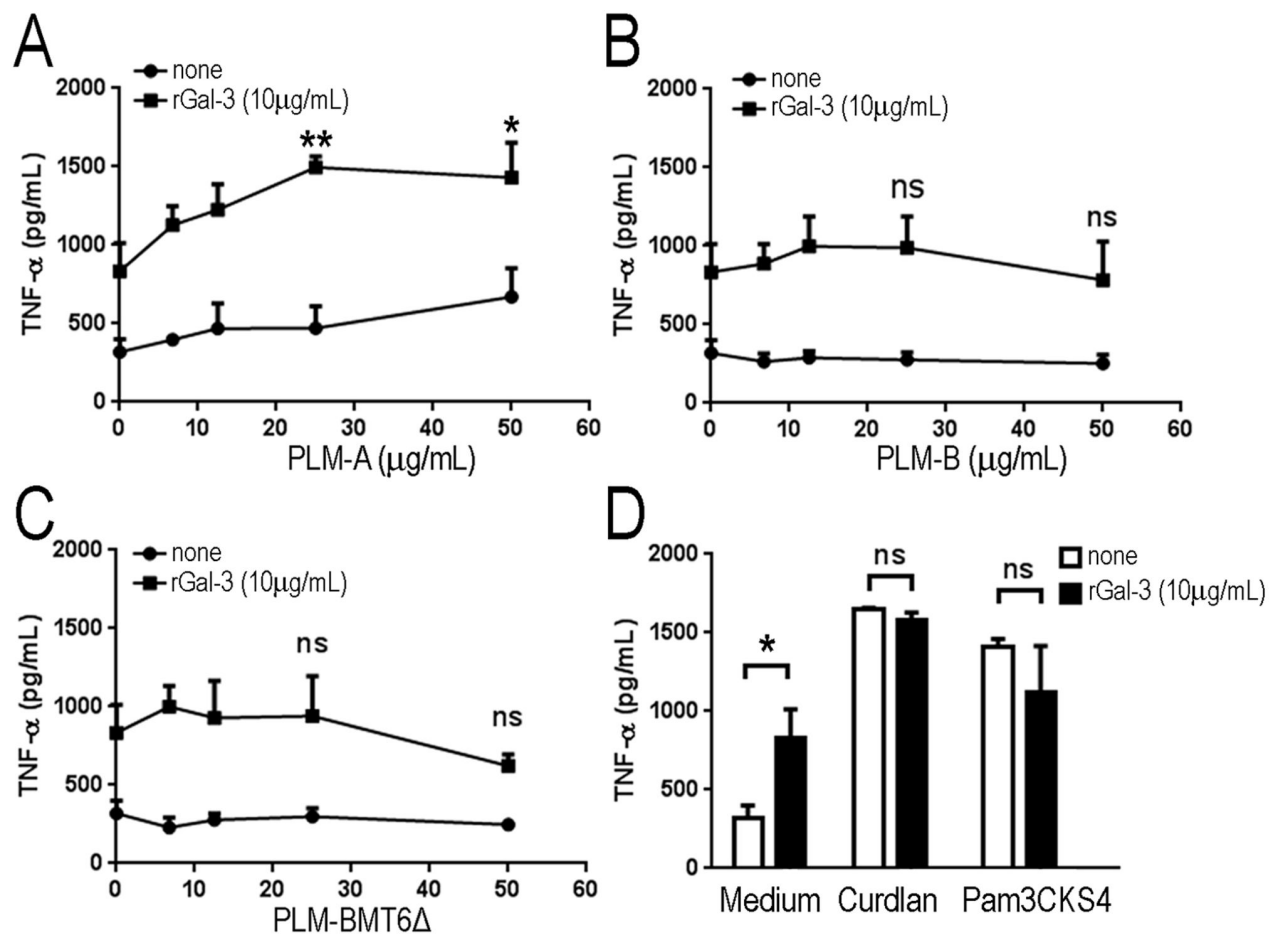
Soluble galectin-3 potentiates PLM-A-induced TNF-α production. Exogenous rGal-3 (10 µg/ml) was preincubated (■) or not (□) with increasing concentrations of PLM-A (A), PLM-B (B) or PLM-BMT6∆ (C) for 1h. The mixtures were then added to J774 macrophages and incubated for an additional 4h. TNF-α concentration in cell-free supernatants was determined by ELISA. (D) Exogenous rGal-3 (10 µg/ml) was preincubated (■) or not (□) with 10 µg/ml of curdlan or 500 ng/ml of Pam3CSK4. Results are represented as the mean ± standard deviation from 3 independent experiments. P values refer to the statistical differences between the effect exerted by galectin-3 alone and in association with PLMs. *p<0.05; **p<0.01.

 Different results were obtained when rGal-3 was preincubated with increasing concentrations of PLMs prior to addition to macrophages (Figure 5). In this case, compared to macrophages stimulated with rGal-3 or PLM-A alone, the complex rGal-3/PLM-A induced a significant increase in TNF-α production by macrophages (Figure 5A). However, different results were obtained with PLM-B and PLM-BMT6∆, both of which did not stimulate TNF-α production even after preincubation with rGal-3 (Figure 5B and 5C, respectively). The addition of lactose, a specific inhibitor of galectin-3, during the preincubation step with rGal-3, decreased but did not abolish the enhancing effect of rGal-3 on PLM-A activity (data not shown). The enhancing effect of rGal-3 on PLM-A activity appears to be specific since no significant effect was observed when curdlan or Pam3CSK4 were preincubated with rGal-3 (Figure 5D). These results demonstrate that soluble galectin-3 may interact with long-chain oligomannosides of PLM-A and potentiates its effects on macrophage activation. 

## Discussion

 The present study was undertaken to examine the role of the glycan moiety of PLM, a glycolipid present in the cell-wall and that plays an important role in the interplay between *C. albicans* and host immune cells [4,6,7,9]. Through a series of experiments, we demonstrated that differential glycosylation of PLM-A, PLM-B and PLM-BMT6∆ does not affect their capacity to induce caspase-1 activation in murine macrophage-like cell line J774. Although each PLM induced the secretion of IL-1β, a higher production was observed after stimulation with PLM-A. Long chains of β-1,2-linked mannosides on PLM are essential for PLM-induced TNF-α since PLMs with short (PLM-B) or truncated (PLM-BMT6∆) β-1,2-linked mannosides were not able to stimulate TNF-α production in macrophages. In addition, long glycan chains on PLM-A favors the formation of complexes with exogenous galectin-3, a glycan-binding protein secreted by macrophages [24] and that binds to β-1,2 mannosylated compounds [11].

 We have previously shown that PLM, a *C. albicans* cell-wall glycolipid bearing β-1,2-linked oligomannosides, induces TNF-α production through an interaction involving galectin-3 and TLR2 [2,11,25]. PLM has been shown to interact with host cells and to induce both pro-inflammatory cytokine production [4,6] and apoptosis through the regulation of ERK signaling pathway via regulation of phosphatase MKP-1 [9]. However, until now, the role of the glycan moiety of PLM in macrophage activation has not been completely investigated since there were no mutant yeasts lacking specific mannosyltransferases required for PLM glycosylation. We recently obtained a series of mutants of *C. albicans* altered in their capability to mannosylate different cell wall components [16]. Among them, *bmt6*∆ mutant was shown to be altered in its ability to elongate the glycan arm of the PLM molecule [17], resulting in a short oligommanosidic moiety similar to one of the major PLM components present in *C. albicans* serotype B [10].

 Recently, it was demonstrated that *C. albicans* triggers activation of the inflammasome [26,27] and, therefore, we hypothesized that PLMs may act as a stimulus that activates the inflammasome in macrophages. Surprisingly, both mannosylated PLM and PLM bearing short chains of oligomannosides were able to induce caspase-1 cleavage, a marker of inflammasome activation. However, PLMs-induced caspase-1 activation is not directly related to the glycosylation status of PLM indicating that the lipid core of PLM, which is similar for all PLMs studied, accounts for inflammasome activation. Accordingly, soluble galectin-3 was not able to potentiate PLMs-induced caspase-1 activation (data not shown).

 Even though PLMs induce the activation of caspase-1 and secondarily the secretion of IL-1β, our results demonstrated that none of the PLMs tested were able to induce ROS production, which suggests that PLM-induced inflammasome activation is mediated by Syk-ROS independent pathway, as opposed to other PAMPs such as beta-glucans [26,28]. Since the lipid moiety is involved in the pro-apoptotic activity of PLM through modulation of MKP-1, a phosphatase related to ERK [29], it is possible that the lipid core of PLM plays a major role in caspase-1 activation, independently of ROS production or phagocytosis. Indeed, the structure of the lipid core of PLMs resembles that of lipids present in ceramides [1] which are known to activate Nlrp3 inflammasome [30]. Experiments are in progress to define the role of the lipid core of PLM in inflammasome activation and the IL-1β secretion processes.

 PLM isolated from *C. albicans* serotype A, whose mannosylated arm is composed of long chains of oligommannosides with up to 19 β-1,2-linked mannoses [1], was able to induce strong TNF-α production in macrophages. In comparison, PLM from *C. albicans* serotype B whose glycan moiety is composed of up to 10 mannoses with predominant trimannosides [10], and PLM from *bmt6*∆ mutant of *C. albicans*, which presents only short chains of oligomannosides with up to three linked mannoses, did not stimulate the cells to produce TNF-α. This is in agreement with our previous results [25], which showed that the activity for β-1,2-linked oligomannosides in macrophages was achieved with chains presenting at least four mannose residues, with optimal activity being observed with chains composed of at least eight β-1,2-linked mannoses. This emphasizes the predominant role of sugars in PLM activity since neither PLM-B nor PLM-BMT6∆ were able to stimulate the cells. We also demonstrated that phagocytosis was not necessary for the PLM-A-induced proinflammatory activity and, therefore, the inhibition of actin polymerization by Latrunculin-A did not modify its properties. This result suggested that long chains of β-1,2-linked mannoses present on PLM-A stimulate TNF-α production by interacting directly with its receptor at the cell membrane. Therefore, enhanced complex formation and endocytosis of PLM displaying incomplete glycosylation do not account for the decreased capability of PLM-B or PLM-BMT6∆ to stimulate macrophages.

 We have previously shown that galectin-3 binds to β-1,2 mannosides from *C. albicans* [11] and is required to discriminate between the pathogenic yeast *C. albicans* from the non-pathogenic *Saccharomyces cerevisiae* [13]. In this study, we demonstrated that soluble rGal-3 potentiates the PLM-induced stimulation of macrophages, as demonstrated for PLM-bearing long chain of β-1,2-linked mannoses (PLM-A). It is not known whether the formation of complexes between PLM and galectin-3 would enhance secondary binding to TLR2 or to other receptors known to interact with galectin-3 and favor recognition of PLM. 

 In contrast to soluble galectin-3, increasing the surface-bound galectin-3 level did not result in any further increase in TNF-α production by macrophages. Galectin-3 has been shown to associate with TLR2 [13] but also with other membrane receptors such as Dectin-1, thus favoring the interaction of the yeasts with the macrophage plasma membrane [31]. Galectin-3 is able to interact with other glycoproteins present at the cell membrane, which could increase the binding of PLM to the plasma membrane receptors. In our experimental settings, we can not rule out that surface-bound galectin-3 may indeed lead to an increase in PLM-binding to the cell surface. However, since no increased stimulation was observed in comparison with PLM-A pretreated with rGal-3, we suggest that soluble galectin-3 may affect PLM complex formation, which leads to enhanced activation. Indeed, it has been recently shown that LPS preincubation with galectin-3 decreases the LPS concentration threshold for neutrophil activation [22].

 The results of this study show that PLM is able to activate inflammasome pathway through a ROS-independent mechanism. This activity seems to be related to the lipid moiety of the molecule since all tested PLMs were able to induce caspase-1 activation and IL-1β secretion. On the other hand, the induction of TNF-α production is dependent on the glycan moiety displayed by PLM; PLMs-bearing short length or truncated β-1,2-linked mannosides were not able to stimulate TNF-α production in macrophages. PLM activity is not influenced by phagocytosis, suggesting that the interaction with receptors occurs at the cell membrane. Galectin-3, a host cell receptor for β-1,2-linked mannoses, enhances PLM-induced TNF-α production and may act as an amplifier of inflammatory response caused by *C. albicans*. Altogether, our results show that depending on the pathway examined, both the lipid core and the glycan moiety of PLM are important for macrophage activation. 
